# Distinct Molecular Mechanisms in Oral Mucosal Wound Healing: Translational Insights and Future Directions

**DOI:** 10.3390/ijms262110660

**Published:** 2025-11-01

**Authors:** Priscila Chuhuaicura, Cynthia Rodríguez-Niklitschek, Gonzalo H. Oporto, Luis A. Salazar

**Affiliations:** 1 Doctoral Programme in Sciences Major in Applied Cellular and Molecular Biology, Universidad de La Frontera, Temuco 4811230, Chile; priscila.chuhuaicura@ufrontera.cl; 2 Department of Integral Adult Dentistry, Oral Biology Research Centre (CIBO-UFRO), Dental School, University of La Frontera, Temuco 4780000, Chile; cynthia.rodriguez@ufrontera.cl (C.R.-N.); gonzalo.oporto@ufrontera.cl (G.H.O.); 3 Center of Molecular Biology and Pharmacogenetics, Scientific and Technological Bioresource Nucleus, University of La Frontera, Temuco 4811230, Chile

**Keywords:** wound healing, oral mucosa, mouth mucosa, signaling pathway

## Abstract

Oral mucosal wound healing is a rapid, precisely regulated process distinct from cutaneous repair due to the specialized anatomical, microbial, and physiological features of the oral cavity. This review outlines the sequential healing phases—hemostasis, inflammation, proliferation, and remodeling—and examines the coordinated roles of keratinocytes, fibroblasts, endothelial cells, and immune cell subsets in tissue restoration. Central molecular pathways, including PI3K/Akt, JAK/STAT, Ras/MAPK, TGF-β/SMAD, and Wnt/β-catenin, along with growth factors such as TGF-β, FGF, EGF, and VEGF, are discussed in relation to their regulatory influence on cell behavior and extracellular matrix dynamics. Unique intraoral factors—namely saliva-derived histatins and a distinct resident microbiota—promote accelerated re-epithelialization and attenuated fibrosis. Systemic conditions such as diabetes, aging, and tobacco exposure are identified as key modulators that compromise repair efficiency. Emerging therapeutic strategies, including stem-cell-based interventions, microbiota modulation, bioengineered scaffolds, and photobiomodulation, offer translational potential to enhance clinical outcomes in oral tissue regeneration.

## 1. Introduction

The oral mucosa is a soft tissue lining of the oral cavity that acts as a barrier against pathogens and mechanical injuries. This protective function plays a crucial role in overall health [1]. The oral mucosa may be categorized into masticatory mucosa, lining mucosa, and specialized mucosa, each serving distinct functions and exhibiting unique structural characteristics. Masticatory mucosa, found in areas such as the gingiva and hard palate, is composed of keratinized stratified squamous epithelium, which provides enhanced durability and resistance to mechanical stress during mastication. In contrast, non-masticatory mucosa, including the buccal and labial mucosa, is primarily non-keratinized and more flexible, allowing for mobility and facilitating specific functions, namely secretion. While non-masticatory mucosa prioritizes adaptability and comfort during activities including swallowing and speech, masticatory mucosa is specialized for functioning under greater pressure and friction. Additionally, specialized mucosa, localized to regions like the tongue, contains unique adaptations for taste sensation [2,3].

When the integrity of the oral mucosa is compromised, a cascade of cellular and molecular events is initiated to facilitate tissue repair [4]. Wounds are defined as disruptions in the normal structure and function of tissues, potentially involving not only the epithelial layer but also extending into the subcutaneous tissue and adjacent anatomical structures, including tendons, muscles, blood vessels, nerves, and bone [5]. In the oral cavity, such injuries may result from various causes, including surgical procedures, traumatic events, infections, and systemic conditions such as diabetes and autoimmune disorders.

Local factors—including vascular supply, moisture levels, and microbial composition—play a critical role in modulating the wound healing process, thereby impacting recovery time and clinical outcomes [6]. Although the oral mucosa exhibits a notable capacity for rapid re-epithelialization and minimal scarring, which are essential for functional restoration, several factors may complicate this process. For example, impaired vascularization can restrict the delivery of essential nutrients and oxygen, thereby exacerbating delays in tissue repair [7]. Furthermore, an elevated microbial burden may increase the risk of infection, promote sustained inflammation, and impede effective wound resolution [1,3].

Moreover, systemic conditions such as diabetes and immunosuppression present additional challenges by impairing immune function and compromising vascular perfusion, both of which substantially hinder the wound repair process [7]. Consequently, a comprehensive understanding of the molecular mechanisms governing oral wound healing is critical for informing clinical practice and for the development of targeted therapeutic strategies aimed at optimizing repair and improving patient outcomes [6]. Wound healing constitutes a highly complex biological process, characterized by coordinated cellular activities, precise gene regulation, and activation of intricate signaling pathways that collectively drive the sequential phases of tissue regeneration [8]. A detailed comprehension of these processes enables researchers to identify the principal molecular mediators that regulate tissue repair, including key signaling molecules, regulatory proteins, and components of the extracellular matrix. This review offers a comprehensive analysis of the cellular and molecular mechanisms underlying oral mucosal wound healing, emphasizing the distinct biological characteristics that differentiate it from cutaneous repair, and examining both internal and external modulators that influence the healing trajectory.

## 2. Histological and Functional Characteristics of Oral Mucosa

**Figure 1:**
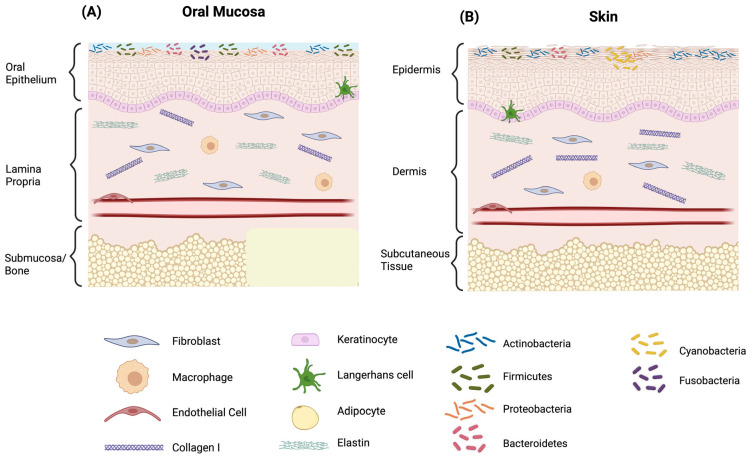
Comparative Features of Oral Mucosa (**A**) and Skin Relevant to Wound Healing (**B**). Comparison of oral mucosa and skin, highlighting key histological and cellular features relevant to wound healing. Both tissues share a stratified squamous epithelium and underlying connective tissue (lamina propria or dermis) composed of fibroblasts, immune cells, and extracellular matrix fibers. Created in BioRender. Chuhuaicura, P. (2025) https://BioRender.com/e3wbbfg (accessed on 29 October 2025).

The oral mucosa comprises a stratified squamous epithelium supported by a lamina propria, with structural and functional variations influenced by anatomical location and mechanical demands (Figure 1). The epithelium includes basal, spinous (prickle), granular, and superficial layers, with basal keratinocytes driving continuous epithelial renewal. The underlying lamina propria harbors fibroblasts, immune cells, endothelial cells, and a collagen-rich extracellular matrix embedded with glycosaminoglycans [9]. Keratinization levels vary, with masticatory regions exhibiting a keratinized epithelium, while lining mucosa remains predominantly non-keratinized. This tissue is characterized by rapid cellular turnover—approximately every 5–8 days in non-keratinized areas and 10–12 days in keratinized regions—facilitating efficient wound closure, limited scar formation, and enhanced regenerative capacity.

In comparison to cutaneous tissue, the oral mucosa displays markedly faster re-epithelialization, a more attenuated inflammatory response, and minimal fibrosis following injury [3]. Despite sharing common cellular constituents, including keratinocytes, fibroblasts, and resident immune cells, the oral mucosa demonstrates superior intrinsic regenerative potential, likely attributed to its unique immune environment and exposure to salivary bioactive factors. As outlined in Table 1, oral mucosal wound healing differs markedly from skin repair in terms of epithelial dynamics, inflammatory resolution, and matrix remodeling, underscoring the distinct molecular and clinical characteristics of intraoral regeneration. This table provides a comparative overview of the fundamental cellular and molecular features distinguishing oral mucosal and cutaneous wound healing. It emphasizes the superior regenerative characteristics of oral tissue. Additional aspects highlighted in the table are explored in detail in the following sections.

## 3. Phases of Oral Mucosal Wound Healing

**Figure 2:**
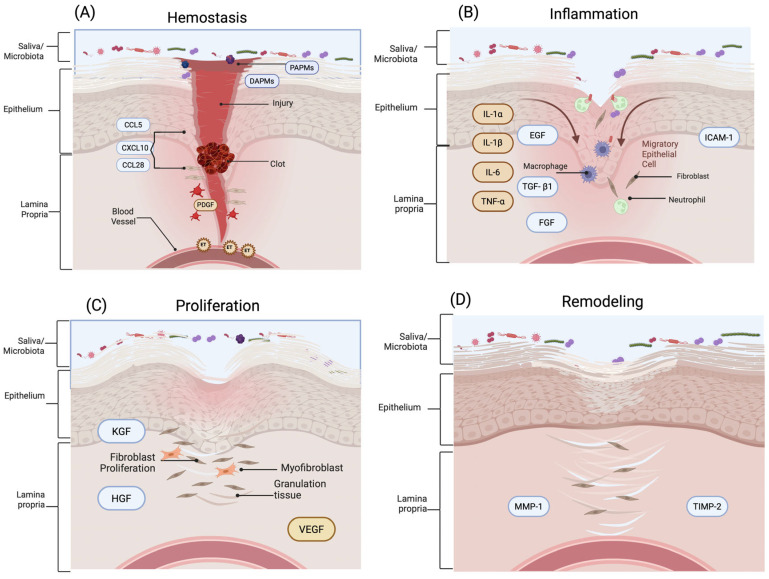
Wound healing process in oral mucosa. (**A**) Hemostasis: Tissue injury triggers vasoconstriction and platelet activation, followed by fibrin clot formation. The release of damage-associated molecular patterns (DAMPs) and pathogen-associated molecular patterns (PAMPs) promotes early immune cell recruitment via chemokines including CCL5, CXCL10, and CCL28, along with platelet-derived growth factor (PDGF) and endothelin-1 (ET). (**B**) Inflammation: Neutrophils and monocyte-derived macrophages infiltrate the wound, secreting pro-inflammatory cytokines (IL-6, IL-1β, IL-1α, TNF-α) and expressing adhesion molecules such as ICAM-1. Growth factors, including epidermal growth factor (EGF), fibroblast growth factor (FGF), and transforming growth factor-β1 (TGF-β1), initiate tissue repair and immune resolution. (**C**) Proliferation: Keratinocytes, fibroblasts, and endothelial cells migrate and proliferate, forming granulation tissue. Chemokines and growth factors—including keratinocyte growth factor (KGF), hepatocyte growth factor (HGF), and vascular endothelial growth factor (VEGF)—drive re-epithelialization and angiogenesis. (**D**) Remodeling: Extracellular matrix (ECM) remodeling occurs through the balanced activity of matrix metalloproteinases (MMPs), such as MMP-1, and tissue inhibitors of metalloproteinases (TIMPs), including TIMP-2, supporting collagen reorganization and restoration of tissue architecture. Created in BioRender. Chuhuaicura, P. (2025) https://BioRender.com/q9df1ec (accessed on 29 October 2025).

Wound healing is a dynamic, highly regulated process that restores tissue integrity after injury, involving coordinated interactions among multiple cell types and signaling molecules [6,10]. This process comprises successive and overlapping phases: hemostasis, inflammation, proliferation, and remodeling (Figure 2). Each one is finely tuned and influenced by the distinct anatomical and physiological characteristics of the oral environment, including constant microbial exposure, rich vascularization, and the presence of saliva [11].

### 3.1. Hemostasis

Hemostasis constitutes the immediate physiological response to tissue injury, initiating a tightly regulated sequence of vascular and cellular events that culminate in the formation of a provisional extracellular matrix essential for subsequent reparative processes [12]. Following vascular disruption, transient vasoconstriction occurs via neurogenic reflexes and the local release of vasoactive mediators such as endothelin-1, effectively minimizing hemorrhage [3]. This is rapidly followed by the adhesion, activation, and aggregation of circulating platelets, resulting in the formation of a primary hemostatic plug. Simultaneously, the coagulation cascade is activated, leading to the generation of thrombin, which catalyzes the conversion of soluble fibrinogen into insoluble fibrin strands [8]. These fibrin polymers integrate with the platelet aggregate to establish a stable, cross-linked fibrin clot that serves as both a mechanical barrier and a bioactive scaffold for cell recruitment. In parallel, the exposure of damage-associated molecular patterns (DAMPs), released from necrotic or stressed host cells, and pathogen-associated molecular patterns (PAMPs), derived from microbial constituents, activates innate immune sensors, thereby initiating the early inflammatory signaling cascade that bridges hemostasis and inflammation [3,12].

In oral mucosal wound healing, the hemostatic response shares core mechanisms with skin, including vascular constriction, platelet activation, and fibrin clot formation. However, the oral cavity presents unique physiological conditions—such as constant moisture, mechanical stress from mastication, and continuous microbial exposure—that influence the efficiency and dynamics of clot stabilization [13]. These conditions necessitate a more rapid and adaptable hemostatic response compared to cutaneous tissue. Understanding these differences is essential for developing targeted therapeutic strategies that optimize hemostatic responses and promote effective oral wound healing, particularly in clinical settings where complications may arise due to the unique physiological conditions of the oral cavity [3,8].

### 3.2. Inflammatory

The inflammatory phase of wound healing involves a tightly regulated immune response aimed at controlling hemorrhage, preventing microbial invasion, and initiating tissue repair. In the oral cavity, this response exhibits distinct characteristics compared to the skin, influenced by the anatomical, vascular, and microbiological uniqueness of the mucosal environment [1,3]. In both tissues, inflammation is triggered by platelet activation and the subsequent release of chemotactic and pro-inflammatory mediators, which recruit immune cells to the site of injury. Neutrophils are the first responders, guided by interleukin-8 (IL-8) and complement-derived peptides, and are responsible for the phagocytosis of cellular debris and pathogens [3,8]. These are followed by monocytes, which differentiate into macrophages under the influence of monocyte chemoattractant protein-1 (MCP-1/CCL2) and granulocyte-macrophage colony-stimulating factor (GM-CSF). Macrophages then coordinate the immune response by releasing key cytokines such as interleukin-1β (IL-1β), interleukin-6 (IL-6), and tumor necrosis factor-alpha (TNF-α), thereby amplifying local inflammation, enhancing vascular permeability, and activating fibroblasts and epithelial progenitor cells [3,14].

In the oral mucosa, the inflammatory phase tends to resolve more rapidly than in skin, largely due to a timely phenotypic switch in macrophages from the pro-inflammatory M1 to the pro-resolving M2 subtype [1,8]. This transition promotes anti-inflammatory signaling, extracellular matrix remodeling, and tissue repair, contributing to reduced fibrosis. Additionally, the oral mucosa’s rapid healing is facilitated by its high epithelial turnover, the presence of salivary growth-promoting factors such as histatins and leptin, and a microbiota capable of modulating local immune responses [1,8,14].

### 3.3. Proliferation

The proliferation phase involves the migration and proliferation of fibroblasts, keratinocytes and endothelial cells, essential for re-epithelialization and the formation of granulation tissue [15]. During this phase, basal keratinocytes at the wound margins are stimulated to proliferate and migrate over the wound bed, orchestrated by growth factors such as epidermal growth factor (EGF), transforming growth factor-α (TGF-α), and interleukin-1β (IL-1β). Fibroblasts within the lamina propria are activated and proliferate, depositing a transient extracellular matrix (ECM) rich in fibronectin and collagen type III, essential for granulation tissue formation. The ECM serves as both a structural scaffold and a biochemical reservoir, guiding tissue regeneration while preventing fibrotic remodeling [2].

Concurrently, endothelial cells initiate angiogenesis through the vascular endothelial growth factor (VEGF)-mediated signaling, ensuring metabolic support to regenerating tissues [8,13]. Angiogenesis is a pivotal component of the proliferative phase in wound healing, ensuring sufficient oxygenation and nutrient delivery to support tissue regeneration [8,16].

In contrast, the proliferation phase in skin wounds typically requires the formation of a scab, which may delay cell migration and regeneration due to the dry environment and the need for additional time for granulation tissue to develop beneath the scab. Furthermore, oral fibroblasts display increased proliferative and migratory potential and differ in their expression of matrix remodeling enzymes, contributing to a more favorable healing environment. These biological distinctions underscore the importance of considering tissue-specific mechanisms in wound management and therapeutic design [15,17].

Oral mucosal tissues exhibit a markedly enhanced proliferative capacity compared to skin, as demonstrated by increased epithelial turnover. Studies such as Basso et al. (2016) highlight the upregulation of proliferation-related genes (PCNA, cyclin D1) and wound-modulating integrins (ITGB1) in oral epithelial cells [15]. Furthermore, Jongjitaree et al. (2022) demonstrated that natural bioactive agents such as Thai propolis can further enhance oral fibroblast proliferation and migration in vitro, in part through antioxidant modulation and ECM protein expression, reinforcing the regenerative potential of the oral microenvironment [11].

### 3.4. Remodeling

The remodeling phase of oral mucosal wound healing involves the transition from provisional granulation tissue to a structurally and functionally mature extracellular matrix (ECM), accompanied by restoration of tissue integrity and mechanical strength [1]. During this stage, fibroblasts shift from a proliferative to a matrix-remodeling phenotype, characterized by reduced cellularity and contractile activity [8]. Central to this remodeling process is the orchestrated activity of matrix metalloproteinases (MMPs)—including MMP-1 (collagenase-1), MMP-2 (gelatinase A), and MMP-9 (gelatinase B)—which degrade fibrillar collagen, gelatin, and other ECM components to permit matrix reorganization. These proteases are tightly regulated by tissue inhibitors of metalloproteinases (TIMPs), particularly TIMP-1 and TIMP-2, which bind MMPs in a 1:1 stoichiometric ratio to modulate proteolytic activity and prevent excessive matrix degradation [13]. The balance between MMPs and TIMPs is thus critical for ensuring appropriate ECM turnover and preventing pathological remodeling.

In oral mucosal wounds, the remodeling phase is characterized by markedly attenuated fibrosis compared to skin. Myofibroblasts—key effectors of wound contraction and fibrotic ECM deposition—are less prevalent in oral tissues [3,8]. This is attributed to diminished transforming growth factor-β1 (TGF-β1) signaling and reduced expression of alpha-smooth muscle actin (α-SMA), limiting fibroblast-to-myofibroblast differentiation [13]. The reduced myofibroblast activity aligns with the scar-minimizing phenotype of oral wounds, resulting in near-complete architectural restoration.

Furthermore, resolution of neovascularization and inflammation occurs more rapidly in oral tissues. Vascular regression is facilitated by a decline in VEGF expression and enhanced pericyte-mediated vessel stabilization [18]. Concurrently, diminished chemokine gradients lead to decreased immune cell infiltration and restoration of tissue homeostasis. Toma et al. (2021) underscore the significance of these tightly controlled dynamics, noting that oral wound models consistently demonstrate expedited recovery and minimal scarring relative to cutaneous wounds [1,3]. Elucidating these molecular and cellular mechanisms holds promise for developing targeted therapeutic strategies aimed at enhancing wound resolution while mitigating fibrosis and infection risk.

## 4. Cellular and Molecular Mechanisms in Oral Healing

### 4.1. Role of Immune Cells

Innate and adaptive immune cells play integral roles in the temporal orchestration of oral mucosal wound healing. Among the earliest responders are neutrophils, which are rapidly recruited to the wound bed in response to damage-associated molecular patterns (DAMPs), chemotactic gradients (e.g., IL-8), and cytokines released by epithelial and endothelial cells [19]. Neutrophils contribute to microbial clearance and the removal of necrotic debris through the release of proteolytic enzymes, reactive oxygen species (ROS), and neutrophil extracellular traps (NETs) [8]. These cells are subsequently cleared via apoptosis and efferocytosis, a critical event that limits collateral tissue damage and facilitates the transition to resolution [1,3].

Macrophages are recruited through CCL2-mediated chemotaxis and originate from both circulating monocytes (CD14^+^CD16^+^) and resident precursors. Upon arrival, they undergo phenotypic polarization in situ, transitioning from a classically activated M1-like state (characterized by CD86^+^, HLA-DR^+^ expression and secretion of TNF-α, IL-1β, and IL-6) to an alternatively activated M2-like phenotype (marked by CD206^+^, CD163^+^ expression and secretion of IL-10, TGF-β1, and TGF-β3) [20]. This phenotypic switch occurs more efficiently in oral mucosa than in cutaneous tissue, promoting tissue remodeling and epithelial proliferation with minimal fibrotic consequences [3,8].

Monocytes (CD14^+^), as precursors of macrophages and dendritic cells, are essential for the replenishment of the local phagocytic pool and modulation of inflammation. Additionally, dendritic cells, including Langerhans cells within the epithelium (CD1a^+^, CD207^+^), contribute to immune surveillance and cytokine production, aiding in the coordination between innate and adaptive responses. T lymphocytes are increasingly recognized as pivotal regulators of mucosal repair. Regulatory T cells (Tregs) modulate excessive inflammation by secreting IL-10 and TGF-β, maintaining immune tolerance and supporting regenerative processes [20]. CD4^+^ helper T cells (Th1 and Th2 subsets) and CD8^+^ cytotoxic T cells also contribute by influencing macrophage polarization and epithelial cell proliferation [1,3]. Pereira et al. [13] have highlighted that the oral mucosa maintains a tolerogenic immune environment due to chronic exposure to commensal microbiota and mechanical stimuli, which primes immune cells for rapid and efficient resolution of inflammation.

Furthermore, the cross-communication between epithelial cells and immune populations is central to orchestrating the healing response. Oral epithelial cells secrete cytokines such as IL-1β and chemokines like IL-8 that guide the recruitment and functional orientation of immune cells [21]. In return, immune-derived signals feedback to modulate epithelial proliferation, migration, and differentiation, as described by Toma et al. [1]. This coordinated cellular interplay, characterized by timely recruitment, phenotypic plasticity, and regulatory balance, underpins the accelerated and minimally scarring phenotype of oral mucosal tissue [3]. Understanding the specific immune cell subsets and their signaling dynamics provides a framework for designing immunomodulatory interventions in tissue repair.

### 4.2. Tissue-Resident Cell Dynamics in Oral Mucosal Repair

Oral mucosal wound healing is characterized by rapid re-epithelialization. These features are primarily linked to the distinctive molecular signatures and context-specific activities of tissue-resident cells. The oral mucosa harbors a distinct population of resident epithelial cells, fibroblasts, and immune cells, whose coordinated response to injury drives efficient repair with attenuated inflammation and fibrosis [3].

Epithelial cells at the wound margins are primed for rapid activation, proliferation, and migration. These cells exhibit heightened basal turnover rates and respond swiftly to injury via upregulation of proliferation-related genes and growth factor receptors such as epidermal growth factor receptor (EGFR), fibroblast growth factor receptor (FGFR). The expression of key mediators such as epidermal growth factor (EGF), transforming growth factor-alpha (TGF-α), and Heparin-binding epidermal growth factor (EGF)-like growth factor (HB-EGF) in both tissue and saliva accelerates wound closure [8,15].

Resident fibroblasts in the oral lamina propria display a transcriptional profile conducive to regenerative healing. They secrete a controlled ECM rich in fibronectin and low-density collagen, supporting keratinocyte migration without excessive matrix stiffness [8,22].

Tissue-resident immune cells, including macrophages and dendritic cells, play a dual role in clearing debris and orchestrating repair. In oral wounds, these cells secrete lower levels of pro-inflammatory cytokines such as interleukin-6 (IL-6) and interleukin-1β (IL-1β) and resolve inflammation more rapidly than in skin wounds [3,22]. Their activity is modulated by the tissue microenvironment and salivary bioactive compounds, such as histatins, mucins, growth factors (e.g., EGF, VEGF), and antimicrobial peptides, which together dampen excessive inflammation and support expedited healing [1,2,3].

Mesenchymal progenitor cells, such as gingiva-derived mesenchymal stem cells (GMSCs), located within the gingival lamina propria and perivascular niches, contribute to oral wound healing by differentiating into multiple lineages and secreting trophic factors that enhance epithelial and fibroblast activity. Their immunomodulatory functions further promote a pro-resolving environment that supports tissue repair while limiting fibrosis [3,8].

## 5. Key Signaling Pathways and Growth Factors

**Figure 3:**
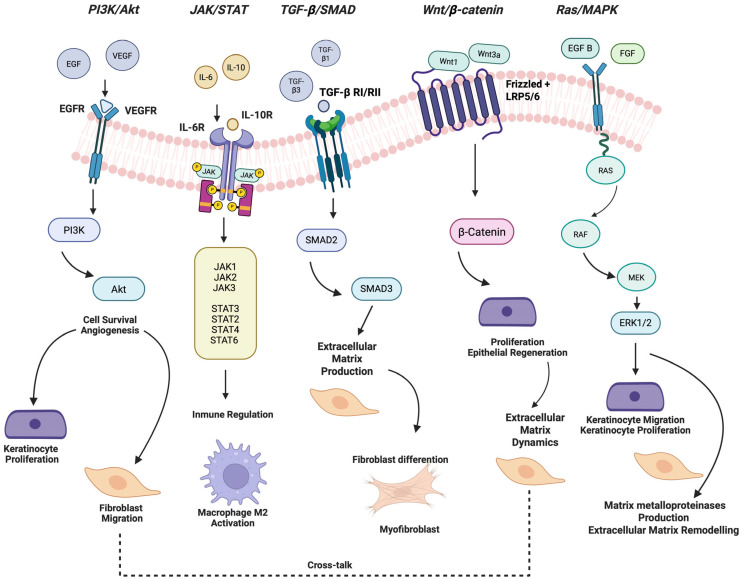
Key signaling pathways and growth factors in oral wound healing. This schematic illustrates the principal intracellular signaling cascades implicated in the regulation of oral mucosal wound healing, including the PI3K/Akt, JAK/STAT, Ras/MAPK, TGF-β/SMAD, and Wnt/β-catenin pathways. These pathways govern essential cellular functions such as proliferation, migration, differentiation, and extracellular matrix remodeling, thereby contributing to tissue regeneration. The diagram also highlights major growth factors—epidermal growth factor (EGF), fibroblast growth factor (FGF), transforming growth factor-β (TGF-β), vascular endothelial growth factor (VEGF)—that act as upstream regulators of these pathways. Arrows indicate activation or promotion of downstream signaling, while dashed lines denote modulatory or cross-talk interactions among pathways. The integration of these signaling networks is critical for the efficient and scarless repair characteristic of oral mucosal tissues. Created in BioRender. Chuhuaicura, P. (2025) https://BioRender.com/kru23jk (accessed on 29 October 2025).

Signal transduction pathways are a series of molecular events that transmit signals from growth factors to the nucleus, regulating various cellular processes. These pathways translate extracellular signals, particularly growth factors and cytokines, into transcriptional and phenotypic changes that guide cellular proliferation, migration, differentiation, and extracellular matrix remodeling (Figure 3). The efficiency and regenerative quality of oral wound repair, compared to cutaneous healing, can be largely attributed to the finetuned regulation of these pathways in tissue-resident cells. The following sections highlight the major signal transduction pathways involved in oral wound healing, supported by both oral-specific and skin-based experimental evidence.

### 5.1. PI3K/Akt Signaling Pathway

The phosphoinositide 3-kinase (PI3K)/Akt axis is a pivotal regulator of cellular processes critical to mucosal repair, including epithelial proliferation, survival, migration, and angiogenesis. In the context of oral wound healing, activation of this pathway is mediated by growth factors such as epidermal growth factor (EGF) and vascular endothelial growth factor (VEGF), which are secreted by resident cells and enriched in saliva [8]. Upon activation, PI3K catalyzes the phosphorylation of phosphatidylinositol-4,5-bisphosphate (PIP2) to generate phosphatidylinositol-3,4,5-trisphosphate (PIP3), recruiting Akt to the plasma membrane where it is phosphorylated and activated. Activated Akt orchestrates multiple downstream signaling events that enhance keratinocyte proliferation, fibroblast migration, and endothelial sprouting, thereby accelerating re-epithelialization and tissue remodeling [23,24].

Experimental data from cutaneous models have demonstrated the ability of PI3K/Akt activation to enhance wound closure through exosome-mediated delivery from adipose-derived stem cells, milk-derived extracellular vesicles, and phytochemicals such as garcinol [25,26,27]. In aged murine models, Tideglusib—a non-EGFR-dependent PI3K/Akt activator—restored epidermal stem cell activity and accelerated re-epithelialization [28].

In oral tissues, comparable molecular mechanisms are observed. Notably, *Lactobacillus reuteri* bacterial extracts have been shown to enhance gingiva-derived mesenchymal stem cell (GMSC) migration, proliferation, and osteogenic differentiation through the PI3K/Akt/β-catenin/TGF-β1 axis. This cascade also upregulates MMP1, MMP2, MMP9, and MMP13 expression, facilitating extracellular matrix remodeling during mucosal repair. The pathway further modulates epithelial–mesenchymal transition (EMT), contributing to the plasticity of basal epithelial cells and promoting regenerative responses characteristic of scarless healing in the oral cavity [23,24].

### 5.2. JAK/STAT Signaling Pathway

The Janus kinase (JAK)/signal transducer and activator of transcription (STAT) pathway encompass multiple isoforms with distinct yet overlapping functions, each selectively engaged by different cytokine receptors [29]. This signaling axis is critical for regulating inflammation, cell proliferation, and immune cell behavior during wound healing. In the oral mucosa, STAT3 activation in keratinocytes and macrophages facilitates the resolution of inflammation and the initiation of repair. Notably, Ko et al. [7] demonstrated that gingiva-derived mesenchymal stem cells enhance oral wound healing by upregulating IL-10 and activating STAT3, thereby promoting anti-inflammatory macrophage polarization toward the M2 phenotype [30].

Pathological studies in oral mucosal lesions have also implicated the JAK/STAT pathway in disease-related epithelial changes. Ociepa et al. reported altered expression of JAK3, STAT2, STAT4, and STAT6 in patients with oral lichen planus, pemphigus vulgaris, and chronic ulcerative stomatitis, suggesting its role in both healing and immune-mediated pathology [30].

In skin wound models, hydrogel-based delivery of the JAK1 inhibitor filgotinib has been used to control inflammatory responses and support angiogenesis. Xie et al. [31] and Chu et al. [32] demonstrated that such delivery systems not only modulate macrophage polarization via STAT3 but also enhance VEGF production and antioxidant defense, significantly improving diabetic wound healing outcomes. These studies collectively underscore the therapeutic relevance of precisely tuning JAK/STAT activity in mucosal and dermal tissues.

### 5.3. Ras/MAPK Signaling Pathway

The Ras/Mitogen-Activated protein kinase (MAPK) pathway, which includes Extracellular signal-regulated kinase 1/2 (ERK1/2), Jun N-terminal kinase (JNK), and p38 MAPK-protein, governs multiple aspects of cellular behavior in response to injury. Upon oral mucosal damage, ERK1/2 signaling is rapidly activated in basal keratinocytes, promoting proliferation and directed migration necessary for re-epithelialization [1,2,3]. Additionally, MAPK pathways influence the expression of pro-inflammatory cytokines and matrix metalloproteinases in fibroblasts, impacting ECM remodeling. Farooq et al. [33] emphasized that the oral environment modulates MAPK activity in a manner that restricts excessive myofibroblast differentiation and inflammation, unlike in dermal wounds, where sustained p38 and JNK activity often leads to fibrosis [33]. The balanced and transient activation of ERK signaling in oral tissues is likely a key contributor to the rapid and scarless healing phenotype [34].

### 5.4. TGF-β1 Signaling Pathway

Transforming growth factor-beta (TGF-β) signaling via SMAD2/3 (homologs of the Drosophila protein ‘Mothers against decapentaplegic’ (MAD) and the Caenorhabditis elegans protein SMA) is a master regulator of tissue repair, particularly in modulating ECM deposition and myofibroblast transdifferentiation [35]. In oral fibroblasts, TGF-β1-induced signaling is notably less robust than in dermal counterparts, resulting in limited myofibroblast formation and reduced contractile activity [7,8,36]. This inherent insensitivity to pro-fibrotic stimuli facilitates scarless regeneration of oral tissues.

In contrast, cutaneous wound healing often involves sustained TGF-β1 signaling, which promotes granulation tissue contraction and collagen cross-linking but also leads to pathological scarring in some cases [36]. Thus, the temporally restricted and spatially localized TGF-β activity observed in oral wounds contributes to a regenerative remodeling of the ECM without fibrosis [13,35,37].

### 5.5. Wnt/β-Catenin Signaling Pathway

The Wnt/β-catenin pathway regulates cell fate determination, stem cell maintenance, and epithelial regeneration. Although direct functional studies in oral wound healing are limited, evidence from oral mucosal models indicates early Wnt activation following injury, promoting basal cell proliferation and re-epithelialization [7]. Thompson et al. 2024 describe some mechanisms of distal epithelial cells in wound healing [38]. This pathway also interacts with TGF-β and PI3K/Akt signaling to modulate fibroblast activity and ECM dynamics. Dysregulation of Wnt signaling in skin models has been associated with delayed healing and fibrosis, underscoring the importance of its tight regulation. In the oral mucosa, the transient and controlled activation of Wnt appears to support regenerative healing while preserving epithelial architecture [39,40,41].

### 5.6. Growth Factors

There are five known superfamilies of growth factors. The growth factors, along with their receptors, vary in structure and cell pathway activation between families and within each family. Still, some consistent exist. Most growth factors originate from large proteins or multiple gene products and undergo posttranslational modification before being released in an active state. These signaling molecules bind to specific receptors on the surface of target cells, which initiate a cascade of intracellular signaling that regulates several physiological functions, including tissue development, wound healing and immune response [42,43].

#### 5.6.1. Fibroblast Growth Factor (FGF)

Fibroblast Growth Factor (FGF) is a family of 22 proteins that regulate various cellular processes, including cell growth, differentiation, and migration. FGFs play a crucial role in embryonic development, tissue repair, and angiogenesis. They are also involved in the regulation of growth factor signaling pathways, making them an important aspect of growth factor regulation [42]. Several members of the FGF family participate in the wound healing process. FGF-1 and FGF-2 are recognized as key regulators in skin wound repair. In animal models, mice deficient in FGF-2 exhibit impaired re-epithelialization and decreased collagen deposition following skin injury, and it has also been reported as an activator not only of fibroblast but also mesoderm-derived cells such as osteoblast, chondrocytes and smooth muscle cells [43,44,45]. In addition, FGF-1 has shown a crucial role in angiogenesis and the wound healing process and participates in various stages of development and morphogenesis [46,47,48]. In oral wound healing, FGF-2 has been found in rats’ saliva but with no significant effects in comparison to skin wound healing; similar levels of FGF-2 expression were also found in primary oral and skin cells [3]. However, other animal models have shown an increased rate of vascularization with a scaffold loaded with FGF-2 in palate repair, as well as a faster influx of host cells [45].

#### 5.6.2. Epidermal Growth Factor (EGF)

Epidermal Growth Factor (EGF) is a protein that plays a crucial role in regulating cell growth, proliferation and differentiation. It is involved in various cellular processes, including wound healing, tissue repair, and embryonic development. EGF is a key regulator of growth factor signaling pathways, making it a vital component in understanding growth factor regulation [48]. In oral wound healing, the use of recombinant human epidermal growth factor (rhEGF) promotes wound healing in oral and maxillofacial (OMF) trauma, exhibits shorter healing times and reduces incision edema duration, and less scar hyperplasia was significantly less frequent in the rhEGF group [49]. Another study suggests [50] that EGF is important as an indicator and facilitator of enhanced tissue remodeling and oral wound healing in rats.

EGF receptors (EGFr), also known as ErbB, are tyrosine kinase receptors mainly expressed on several cell types. They activate a network of signaling pathways that is tightly regulated by phosphorylation, subcellular localization of receptors, and formation of protein complexes regulating migration, proliferation, and cell differentiation [51]. Some studies have reported that curcumin upregulates EGFR expression in both unwounded human gingival fibroblasts (hGFs) in an in vitro model. The upregulation of EGFR by curcumin in the wound healing model is dependent on ERK signaling, suggesting a mechanistic pathway by which curcumin may promote oral wound healing [52]. Other authors have observed significantly higher expression levels of EGFR in the treatment groups compared to the control group, suggesting accelerated re-epithelialization in skin [53].

#### 5.6.3. Platelet-Derived Growth Factor (PDGF)

Platelet-Derived Growth Factor (PDGF) is a protein that regulates cell growth, proliferation and migration [54]. It is involved in various cellular processes, including wound healing, tissue repair, and embryonic development. PDGF is a key regulator of growth factor signaling pathways, making it an essential component in understanding growth factor regulation. In skin wounds, this factor promotes the migration of keratinocytes, ensuring the formation of a skin barrier [55]. Along with IL-1, PDGF attracts neutrophils to the wound site to remove contaminating bacteria [56]. Furthermore, clinical evidence suggests that recombinant human platelet-derived growth factor (rhPDGF) is a safe and effective adjunct for treating infrabony and furcation periodontal defects, gingival recession, and guide bone regeneration and alveolar ridge preservation [57].

#### 5.6.4. Vascular Endothelial Growth Factor (VEGF)

Vascular Endothelial Growth Factor (VEGF) is a protein that regulates angiogenesis, the formation of new blood vessels from pre-existing ones. VEGF is involved in various cellular processes, including wound healing, tissue repair, and embryonic development [58]. It is a key regulator of growth factor signaling pathways, making it a vital component in understanding growth fac-tor regulation. VEGF increases vascular permeability, allowing plasma proteins like fibrinogen to leak into the wound area and form a fibrin matrix scaffold that facilitates epithelial cell migration [59,60]. While VEGF is essential for angiogenesis, oral mucosal wounds exhibit less vascular proliferation than skin wounds despite faster healing. This paradox is linked to downregulation of VEGF receptors (VEGFR-1/VEGFR-2) in oral mucosa, which may prevent excessive vascularization and scarring [60]. Saliva is a primary source of VEGF in oral wounds, directly influencing mucosal repair. Selective removal of submandibular glands (which produce VEGF-rich saliva) in mice reduced wound closure rates by 40% and impaired neovascularization. Oral VEGF supplementation restored normal healing [61].

#### 5.6.5. Transforming Growth Factor-Beta (TGF-β)

Transforming Growth Factor-beta (TGF-β), particularly the TGF-β1 isoform, is a pivotal cytokine in the regulation of wound healing, exerting complex and context-dependent effects on inflammation, cell proliferation, ECM deposition, and fibrosis [62]. In oral mucosal repair, TGF-β signaling is activated transiently and locally, enabling efficient tissue regeneration while avoiding the excessive fibrotic remodeling characteristic of dermal healing [63].

TGF-β is secreted in a latent form by platelets, epithelial cells, and fibroblasts early after injury. Upon activation, it binds to TGF-β receptors and initiates intracellular signaling primarily through the SMAD2/3 pathway, modulating gene expression in target cells. In fibroblasts, TGF-β induces the production of ECM components such as collagen types I and III, fibronectin, and proteoglycans, and can drive myofibroblast differentiation [3,8].

This attenuated fibrotic response is a key determinant of the scarless phenotype observed in oral wounds [63]. In addition, epithelial cells in the oral mucosa respond to TGF-β by enhancing migration and wound re-epithelialization, while immune cells modulate their inflammatory output under TGF-β control, supporting a rapid resolution phase [62]. Ko et al. (2021) further noted that gingiva-derived mesenchymal stem cells influence TGF-β activity by secreting regulatory cytokines that balance pro-healing and anti-fibrotic signals [7].

#### 5.6.6. Keratinocyte Growth Factor (KGF)

Keratinocyte Growth Factor (KGF), also known as FGF7, is a paracrine-acting member of the fibroblast growth factor (FGF) family, predominantly produced by mesenchymal cells such as fibroblasts [52]. In the context of oral mucosal wound healing, KGF plays a pivotal role in re-epithelialization by promoting proliferation, migration, and differentiation of basal keratinocytes.

KGF binds to its specific receptor, FGFR2b, which is expressed on epithelial cells, activating intracellular cascades such as the Ras/MAPK and PI3K/Akt pathways. These downstream signals enhance keratinocyte survival and mitotic activity, facilitating rapid coverage of the wound bed and restoration of the epithelial barrier. In oral tissues, this process is especially efficient due to the elevated proliferative capacity and stemness of oral keratinocytes compared to their dermal counterparts [7,8].

Furthermore, KGF influences the expression of genes involved in epithelial integrity, including those encoding cytokeratins and adhesion molecules, and it contributes to a non-fibrotic healing environment by stimulating epithelial renewal without excessive ECM deposition or myofibroblast activation. This feature aligns with the minimal scarring observed in oral wounds [64]. Experimental evidence from oral models demonstrates that gingival fibroblasts secrete KGF in response to injury, and this secretion is modulated by inflammatory signals such as IL-1β and TNF-α, establishing a link between immune regulation and epithelial repair [3].

## 6. Modulating Factors in Oral Wound Healing

### 6.1. Microbiota

The oral cavity harbors a diverse and dynamic microbiota comprising over 700 microbial species, including bacteria, fungi, and viruses, that collectively contribute to maintaining mucosal homeostasis. In the context of wound healing, this microbiota functions both as a beneficial modulator and, under dysbiotic conditions, as a pathological disruptor of tissue repair [65,66]. Commensal species—such as *Streptococcus mitis*—have been shown to reinforce epithelial barrier function and regulate immune tolerance through the production of short-chain fatty acids (SCFAs) and interaction with pattern recognition receptors (PRRs) [65,67,68].

Conversely, shifts toward dysbiosis, typically marked by overrepresentation of pathobionts like *Porphyromonas gingivalis*, *Fusobacterium nucleatum*, *Treponema denticola*, and *Candida albicans*, are associated with impaired wound healing, sustained inflammation, and degradation of tissue architecture. These species interfere with key repair processes—including keratinocyte migration, extracellular matrix remodeling, and angiogenesis—via the secretion of proteolytic enzymes, biofilm formation, and lipopolysaccharide-mediated immune dysregulation [23,66,69].

Emerging evidence from in vitro co-culture models supports the notion that increased microbial load and reduced microbial diversity, particularly in mucositis-associated samples, significantly impair epithelial monolayer closure and delay wound healing—even in the absence of chemotherapeutic stressors like 5-fluorouracil [61]. In contrast, in vivo studies have demonstrated that oral application of lysates derived from *Lacticaseibacillus rhamnosus* and *Lacticaseibacillu reuteri* accelerates wound closure and enhances epithelial regeneration, with minimal inflammatory infiltration, highlighting the regenerative potential of non-viable probiotic derivatives [69,70].

Furthermore, the oral virome, including latent viruses such as herpes simplex virus type 1 (HSV-1), Epstein–Barr virus (EBV), and human papillomavirus (HPV), has emerged as an additional modulating factor. These viruses can alter cytokine signaling, delay epithelial migration, and potentially contribute to malignant transformation in chronically injured mucosa, emphasizing the need for microbial surveillance in wound healing protocols [71].

Taken together, the oral microbiota profoundly influences each phase of mucosal wound healing by modulating the inflammatory environment, shaping epithelial-immune cross-talk, and either facilitating or impairing re-epithelialization and matrix remodeling [65,66,68,69,72].

### 6.2. Saliva

Saliva plays a pivotal role in promoting wound healing within the oral cavity due to its rich composition of growth factors, antimicrobial peptides, enzymes, and immunoregulatory molecules. Among its bioactive constituents, histatins, particularly Histatin-1 and Histatin-5, are of notable importance [3,71]. These cationic peptides exhibit potent antimicrobial properties and promote wound healing by enhancing epithelial cell migration, stimulating angiogenesis, and modulating inflammatory responses. Histatin-1 has been shown to activate integrin-mediated adhesion and the MAPK/ERK signaling cascade, thereby facilitating keratinocyte motility and spreading. Histatin-5, in addition to its antifungal activity, has been implicated in promoting endothelial cell proliferation and migration [73,74].

Salivary growth factors such as epidermal growth factor (EGF), transforming growth factor-β (TGF-β), and vascular endothelial growth factor (VEGF) further contribute to the regenerative process by stimulating epithelial proliferation, modulating fibroblast activity, and supporting neovascularization. Tissue factor and fibrinogen present in saliva aid in clot formation and stabilization during the hemostatic phase [75].

Experimental studies have demonstrated that human saliva enhances both dermal and oral fibroblast proliferation and accelerates epithelial closure in in vitro models [15]. Additionally, saliva contributes to tissue hydration and buffering, creating an optimal microenvironment for cellular migration and matrix remodeling. Its constant interaction with the mucosa allows for dynamic regulation of local immunity and repair mechanisms, which likely underpins the accelerated and scarless healing observed in oral tissues.

### 6.3. Vascularization

The establishment of a functional vascular network is fundamental to successful wound healing, as it enables the delivery of oxygen, nutrients, immune cells, and signaling molecules to the regenerating tissue. In oral mucosal wounds, angiogenesis is initiated early during the proliferative phase and proceeds with remarkable efficiency compared to cutaneous wounds. This process is tightly regulated by a cascade of pro-angiogenic signals, predominantly mediated by vascular endothelial growth factor (VEGF), fibroblast growth factors (FGFs), and angiopoietins. These growth factors activate endothelial cells, promoting their proliferation, migration, and alignment into nascent capillary structures [76].

VEGF, secreted by keratinocytes, fibroblasts, and infiltrating macrophages in response to hypoxia and inflammatory cues, acts through VEGF receptors (VEGFR-1 and VEGFR-2) to initiate angiogenic sprouting. Concurrently, FGF-2 (basic FGF) enhances endothelial cell survival and supports the deposition of extracellular matrix (ECM) components necessary for vessel stabilization. Waasdorp et al. (2021) demonstrated that oral wounds exhibit a highly orchestrated endothelial activation profile, with efficient recruitment of pericytes that stabilize neovessels and prevent aberrant angiogenesis [3]. This balance ensures adequate vascular supply without promoting pathological remodeling.

Additionally, oral fibroblasts contribute to the angiogenic niche by producing matrix metalloproteinases (MMPs), particularly MMP-2 and MMP-9, which facilitate ECM remodeling and enable capillary sprouting. The resolution of angiogenesis is equally critical; as the wound closes, anti-angiogenic factors such as thrombospondin-1 and transforming growth factor-β (TGF-β) downregulate neovessel formation, promoting vascular pruning and tissue normalization [77,78].

The oral mucosa’s angiogenic response is thus characterized by rapid onset, effective vascular maturation, and timely regression—features that contribute significantly to its superior regenerative outcomes. A deeper understanding of these processes holds promise for therapeutic modulation of angiogenesis in impaired healing conditions, such as diabetic ulcers and postsurgical complications.

### 6.4. Age

Aging impairs wound healing through cellular senescence, diminished proliferative capacity, and altered immune function. Aged keratinocytes and fibroblasts show reduced responsiveness to mitogenic signals such as EGF and IGF-1. Ko et al. (2021) described a shift in macrophage polarization toward a pro-inflammatory M1 phenotype in aged oral tissues, resulting in delayed inflammation resolution and impaired re-epithelialization [7]. These factors collectively lead to slower healing and increased risk of tissue breakdown. Age is a critical determinant of oral wound healing efficacy. In elderly individuals, the regenerative capacity of epithelial and stromal cells declines, leading to delayed re-epithelialization and reduced angiogenesis. Aging is also associated with impaired immune function and increased oxidative stress, both of which negatively affect the inflammatory and proliferative phases of healing. Consequently, wound closure is slower, and the risk of infection or fibrosis is higher in aged oral tissues [79,80].

### 6.5. Diabetes Mellitus

Diabetes mellitus markedly impairs oral wound healing due to chronic hyperglycemia, which alters vascular integrity, immune responses, and fibroblast function. Diabetic conditions promote prolonged inflammation, delayed collagen deposition, and reduced angiogenesis. Moreover, the formation of advanced glycation end-products (AGEs) and increased oxidative stress disrupt the balance of pro- and anti-inflammatory mediators, leading to chronic wounds and higher susceptibility to infection [19]. Diabetes mellitus is associated with prolonged hyperglycemia and systemic inflammation that negatively affect all stages of wound healing. Tariq et al. (2022) observed impaired fibroblast function, reduced angiogenic signaling (e.g., VEGF), and compromised PI3K/Akt and TGF-β/SMAD pathways in diabetic oral wounds [81]. Furthermore, salivary dysfunction in diabetic patients may exacerbate epithelial damage and microbial imbalance, contributing to chronic wound development [81,82,83].

### 6.6. Tobacco Use

Tobacco smoking is a major extrinsic factor known to compromise oral wound healing. Nicotine and other toxins in tobacco smoke induce vasoconstriction, reduce oxygen tension, and impair neutrophil and macrophage function [84]. These effects hinder hemostasis, inflammation resolution, and tissue regeneration. Additionally, smoking promotes microbial imbalance and reduces salivary flow, further exacerbating the wound environment. Some evidence consistently demonstrates delayed healing and increased postoperative complications in smokers undergoing oral surgical procedures [79]. Tobacco impairs oral wound healing by causing vasoconstriction, oxidative stress, and cytotoxic effects on keratinocytes and fibroblasts. Rathinavelu et al. (2022) reported that nicotine suppresses keratinocyte migration and angiogenesis while altering the salivary proteome and microbiota [85]. These effects lead to delayed wound closure, persistent inflammation, and higher risk of scarring or wound dehiscence [86].

## 7. Clinical Implications and Future Directions

A comprehensive understanding of the cellular and molecular mechanisms underlying oral mucosal wound healing provides a foundation for translating basic science into advanced clinical practice. The oral mucosa exhibits a distinctive regenerative profile compared to cutaneous tissue, characterized by accelerated re-epithelialization, attenuated inflammatory responses, and reduced fibrosis. These intrinsic properties underscore the potential for developing targeted therapeutic interventions, particularly for complex clinical scenarios such as post-extraction sites, mucogingival surgeries, oral grafts, chronic ulcers, and healing in medically compromised patients.

Emerging therapeutic strategies hold significant translational promise for enhancing intraoral tissue regeneration. Stem-cell-based therapies, especially those utilizing gingiva-derived mesenchymal stem cells (GMSCs), have garnered attention due to their potent immunomodulatory properties, high proliferative capacity, and secretion of trophic factors that promote epithelial regeneration, angiogenesis, and extracellular matrix remodeling. Preclinical studies have demonstrated the ability of GMSCs to accelerate wound closure and reduce fibrotic outcomes, supporting their application in regenerative oral medicine [8,87].

Modulation of the oral microbiota represents another frontier in wound management. A balanced microbiome supports immune homeostasis and tissue repair, while dysbiosis may prolong inflammation and impair re-epithelialization [72]. Probiotics such as *L. reuteri* have shown potential in restoring microbial equilibrium, enhancing epithelial integrity, and modulating local cytokine profiles. These interventions are especially relevant in patients with systemic diseases or conditions that predispose to microbial imbalance [23].

Bioengineered scaffolds and smart biomaterials have evolved to not only provide structural support but also deliver bioactive molecules in a temporally controlled manner [1]. Innovations such as hydrogel-based platforms, 3D-printed constructs and nanofiber matrices embedded with growth factors (e.g., FGF, VEGF, PDGF) or stem cells have demonstrated efficacy in enhancing cell migration, vascularization, and tissue remodeling in both experimental and clinical contexts [88].

Photobiomodulation therapy (PBMT), employing low-level laser or light-emitting diode (LED) technology, offers a non-invasive adjunct capable of stimulating mitochondrial function, promoting fibroblast proliferation, and reducing oxidative stress. Clinical applications of PBMT in oral wound healing include its use in accelerating healing post-surgery, managing chronic mucosal lesions, and alleviating pain and inflammation [89,90].

To maximize the translational potential of these therapies, future research must focus on elucidating the precise molecular interactions governing the wound microenvironment, identifying predictive biomarkers of healing capacity, and designing personalized treatment strategies. With the global rise in aging populations and metabolic disorders such as diabetes—both of which compromise wound healing—there is a pressing need to tailor regenerative interventions to individual patient profiles.

Moreover, integration of omics technologies, such as transcriptomics and proteomics, could further refine therapeutic targeting and facilitate the development of precision medicine approaches in oral wound care. Ultimately, translating these scientific advances into clinical protocols will not only enhance outcomes in oral health but also contribute meaningfully to the broader discipline of regenerative medicine.

## Figures and Tables

**Table 1:** Comparative Summary of Oral Mucosa and Skin Wound Healing: Cellular and Molecular Features.

Feature	Oral Mucosa	Skin
Epithelial Turnover	Rapid (5–12 days); high regenerative potential	Slower (28–40 days); limited regenerative capacity
Inflammatory Response	Attenuated and shorter; rapid macrophage M1 to M2 transition	Prolonged and intense; higher risk of chronic inflammation
Myofibroblast Presence	Reduced; low α-SMA expression; minimal fibrosis	Abundant; promotes scar formation
Angiogenesis Dynamics	Rapid, transient, VEGF-regulated; early vascular regression	Delayed; persistent neovascularization
Matrix Remodeling	Balanced MMP/TIMP activity; efficient ECM restoration	Imbalanced; risk of excessive matrix deposition
Microbiota Interaction	Commensals modulate immunity; high microbial diversity	Lower microbial load; less direct influence
Scarring	Minimal to none	Common; visible fibrotic scarring
Clinical Healing Outcome	Faster recovery, improved aesthetics and function	Slower healing, higher risk of complications

## Data Availability

No new data were created or analyzed in this study. Data sharing is not applicable to this article.

## Abbreviations

The following abbreviations are used in this manuscript:

Akt	Protein Kinase B
EGF	Epidermal Growth Factor
EGFR	Epidermal Growth Factor Receptor
ECM	Extracellular Matrix
FGF	Fibroblast Growth Factor
FGF-1	Fibroblast Growth Factor 1
FGF-2	Fibroblast Growth Factor 2
FGF-7	Fibroblast Growth Factor 7 (Keratinocyte Growth Factor)
FGF21	Fibroblast Growth Factor 21
GSH	Glutathione
IL-1β	Interleukin-1 Beta
ITGA5	Integrin Alpha-5
JAK	Janus Kinase
JAK/STAT	Janus Kinase/Signal Transducer and Activator of Transcription Pathway
KGF	Keratinocyte Growth Factor
LLLT	Low-Level Light Therapy
MAPK	Mitogen-Activated Protein Kinase
MEK	Mitogen-Activated Protein Kinase Kinase
MEVs	Milk-Derived Extracellular Vesicles
NF-κB	Nuclear Factor Kappa B
PI3K	Phosphatidylinositol 3-Kinase
STAT	Signal Transducer and Activator of Transcription
TGF-β	Transforming Growth Factor Beta
VEGF	Vascular Endothelial Growth Factor
VEGFR2	Vascular Endothelial Growth Factor Receptor 2

## References

[1] Toma A.I., Fuller J.M., Willett N.J., Goudy S.L. (2021). Oral wound healing models and emerging regenerative therapies. Transl. Res..

[2] Iglesias-Bartolome R., Uchiyama A., Molinolo A.A., Abusleme L., Brooks S.R., Callejas-Valera J.L., Edwards D., Doci C., Asselin-Labat M.-L., Onaitis M.W. (2018). Transcriptional signature primes human oral mucosa for rapid wound healing. Sci. Transl. Med..

[3] Waasdorp M., Waasdorp M., Krom B.P., Bikker F.J., van Zuijlen P.P.M., Niessen F.B., Gibbs S. (2021). The bigger picture: Why oral mucosa heals better than skin. Biomolecules.

[4] de Oliveira Gonzalez A.C., Costa T.F., de Araújo Andrade Z., Medrado A.R.A.P. (2016). Wound healing—A literature review. An. Bras. Dermatol..

[5] Velnar T., Bailey T., Smrkolj V. (2009). The wound healing process: An overview of the cellular and molecular mechanisms. J. Int. Med. Res..

[6] Rodrigues M., Kosaric N., Bonham C.A., Gurtner G.C. (2019). Wound healing: A cellular perspective. Physiol. Rev..

[7] Ko K.I., Sculean A., Graves D.T. (2021). Diabetic wound healing in soft and hard oral tissues. Transl. Res..

[8] Griffin M.F., Fahy E.J., King M., Guardino N., Chen K., Abbas D.B., Lavin C.V., Diaz Deleon N.M., Lorenz H.P., Longaker M.T. (2022). Understanding scarring in the oral mucosa. Adv. Wound Care.

[9] Senel S. (2021). An Overview of Physical, Microbiological and Immune Barriers of Oral Mucosa. Int. J. Mol. Sci..

[10] Trinh X.T., Long N.V., Van Anh L.T., Nga P.T., Giang N.N., Chien P.N., Nam S.Y., Heo C.Y. (2022). A Comprehensive Review of Natural Compounds for Wound Healing: Targeting Bioactivity Perspective. Int. J. Mol. Sci..

[11] Jongjitaree S., Koontongkaew S., Niyomtham N., Yingyongnarongkul B.E., Utispan K. (2022). The oral wound healing potential of Thai propolis based on its antioxidant activity and stimulation of oral fibroblast migration and proliferation. Evidence-Based Complement. Altern. Med..

[12] Hakim R.F., Idroes R., Hanafiah O.A., Ginting B., Kemala P., Fakhrurrazi F., Putra N.I., Shafira G.A., Romadhoni Y., Destiana K. (2023). Characterization of red algae (*Gracilaria verrucosa*) on potential application for topical treatment of oral mucosa wounds in Rattus norvegicus. Narra J..

[13] Pereira D., Sequeira I. (2021). A scarless healing tale: Comparing homeostasis and wound healing of oral mucosa with skin and oesophagus. Front. Cell Dev. Biol..

[14] Overmiller A.M., Sawaya A.P., Hope E.D., Morasso M.I. (2022). Intrinsic Networks Regulating Tissue Repair: Comparative Studies of Oral and Skin Wound Healing. Cold Spring Harb. Perspect. Biol..

[15] Basso F.G., Soares D.G., Pansani T.N., Cardoso L.M., Scheffel D.L., de Souza Costa C.A., Hebling J. (2016). Proliferation, migration, and expression of oral-mucosal-healing-related genes by oral fibroblasts receiving low-level laser therapy after inflammatory cytokines challenge. Lasers Surg. Med..

[16] Rodrigues Neves C., Buskermolen J., Roffel S., Waaijman T., Thon M., Veerman E., Gibbs S. (2019). Human saliva stimulates skin and oral wound healing in vitro. J. Tissue Eng. Regen. Med..

[17] Seeger M.A., Paller A.S. (2015). The Roles of Growth Factors in Keratinocyte Migration. Adv. Wound Care.

[18] Konyaeva A.D., Varakuta E.Y., Leiman A.E., Bolbasov E.N., Chernova U.V. (2023). The Specifics of Neovascularization of Wound Defects in the Oral Mucosa during Its Regeneration under a Piezoelectric Polymer Membrane. Bull. Exp. Biol. Med..

[19] Yadu N., Singh M., Singh D., Keshavkant S. (2025). Mechanistic insights of diabetic wound: Healing process, associated pathways and microRNA-based delivery systems. Int. J. Pharm..

[20] Wang L., Yang K., Xie X., Wang S., Gan H., Wang X., Wei H. (2025). Macrophages as Multifaceted Orchestrators of Tissue Repair: Bridging Inflammation, Regeneration, and Therapeutic Innovation. J. Inflamm. Res..

[21] Basso F.G., Pansani T.N., Turrioni A.P., Soares D.G., de Souza Costa C.A., Hebling J. (2016). Tumor Necrosis Factor-α and Interleukin (IL)-1β, IL-6, and IL-8 Impair In Vitro Migration and Induce Apoptosis of Gingival Fibroblasts and Epithelial Cells, Delaying Wound Healing. J. Periodontol..

[22] Moretti L., Stalfort J., Barker T.H., Abebayehu D. (2022). The interplay of fibroblasts, the extracellular matrix, and inflammation in scar formation. J. Biol. Chem..

[23] Castilho R.M., Squarize C.H., Gutkind J.S. (2013). Exploiting PI3K/mTOR signaling to accelerate epithelial wound healing. Oral Dis..

[24] Han N., Jia L., Su Y., Du J., Guo L., Luo Z., Liu Y. (2019). *Lactobacillus reuteri* extracts promoted wound healing via PI3K/AKT/β-catenin/TGFβ1 pathway. Stem Cell Res. Ther..

[25] Zhang Y., Zouboulis C.C., Xiao Z. (2024). Exosomes from adipose-derived stem cells activate sebocytes through the PI3K/AKT/SREBP1 pathway to accelerate wound healing. Cell Tissue Res..

[26] Fan L., Ma X., Liu B., Yang Y., Yang Y., Ren T., Li Y. (2023). Antioxidant engineered milk-derived extracellular vesicles for accelerating wound healing via regulation of the PI3K/Akt signaling pathway. Adv. Healthc. Mater..

[27] Li Z., Lin K., Wang Y., Mao J., Yin Y., Li Z., Wang F., Zeng X., Li Q., Wang X. (2025). Garcinol promotes wound healing in diabetic mice by regulating inflammation and NLRP3 inflammasome mediated pyroptosis via the PI3K/Akt/NFκB pathway. Int. Immunopharmacol..

[28] Sun J., Zhao H., Shen C., Li S., Zhang W., Ma J., Li Z., Zhang M., Yang J. (2022). Tideglusib promotes wound healing in aged skin by activating PI3K/Akt pathway. Stem Cell Res. Ther..

[29] Jere S.W., Abrahamse H., Houreld N.N. (2017). The JAK/STAT signaling pathway and photobiomodulation in chronic wound healing. Cytokine Growth Factor. Rev..

[30] Ociepa K., Danilewicz M., Wągrowska-Danilewicz M., Peterson-Jęckowska R., Wójcicka-Rubin A., Lewkowicz N., Zajdel R., Żebrowska A. (2023). Expression of the selected proteins of JAK/STAT signaling pathway in diseases with oral mucosa involvement. Int. J. Mol. Sci..

[31] Xie J., Huang Y., Hu X., Wu X., Luo X., Wei P., Jing W., Zhao B., Su J. (2025). A constant Filgotinib delivery adhesive platform based on polyethylene glycol (PEG) hydrogel for accelerating wound healing via restoring macrophage mitochondrial homeostasis. Small.

[32] Chu L., Shen J.-M., Xu Z., Huang J., Ning L., Feng Z., Jiang Y., Wu P., Gao C., Wang W. (2025). Stimuli responsive hydrogel with spatiotemporal co-delivery of FGF21 and H_2_S for synergistic diabetic wound repair. J. Control. Release.

[33] Farooq M., Khan A.W., Kim M.S., Choi S. (2021). The Role of Fibroblast Growth Factor (FGF) Signaling in Tissue Repair and Regeneration. Cells.

[34] Phimnuan P., Dirand Z., Tissot M., Worasakwutiphong S., Sittichokechaiwut A., Grandmottet F., Viyoch J., Viennet C. (2023). Beneficial Effects of a Blended Fibroin/Aloe Gel Extract Film on the Biomolecular Mechanism(s) via the MAPK/ERK Pathway Relating to Diabetic Wound Healing. ACS Omega.

[35] Fang C.Y., Yu C.C., Liao Y.W., Hsieh P.L., Lu M.Y., Lin K.C., Wu C.Z., Tsai L.L. (2019). LncRNA LINC00974 Activates TGF-β/Smad Signaling to Promote Oral Fibrogenesis. J. Oral Pathol. Med..

[36] Zhang S., Elbs-Glatz Y., Tao S., Li Y., Wu Y., Ma Y., Lu Q., Wang Z. (2025). Probiotics Promote Cellular Wound Healing Responses by Modulating the PI3K and TGF-β/Smad Signaling Pathways. Cell Commun. Signal..

[37] Zhang T., Wang X.F., Wang Z.C., Lou D., Fang Q.Q., Hu Y.Y., Zhao W.Y., Zhang L.Y., Wu L.H., Tan W.Q. (2020). Current Potential Therapeutic Strategies Targeting the TGF-β/Smad Signaling Pathway to Attenuate Keloid and Hypertrophic Scar Formation. Biomed. Pharmacother..

[38] Thompson T., Flanagan S., Ortega-Gonzalez D., Zhu T., Yuan X. (2024). Immediate but Temporal Response: The Role of Distal Epithelial Cells in Wound Healing. Stem Cell Rev. Rep..

[39] Barrientos S., Stojadinovic O., Golinko M.S., Brem H., TomicCanic M. (2008). Perspective article: Growth factors and cytokines in wound healing. Wound Repair. Regen..

[40] Yuan X., Xu Q., Zhang X., Van Brunt L.A., Ticha P., Helms J.A. (2019). Wnt-Responsive Stem Cell Fates in the Oral Mucosa. iScience.

[41] Gumede D.B., Abrahamse H., Houreld N.N. (2024). Targeting Wnt/β-catenin signaling and its interplay with TGFβ and Notch signaling pathways for the treatment of chronic wounds. Cell Commun. Signal..

[42] Takaya K., Aramaki Hattori N., Sakai S., Okabe K., Asou T., Kishi K. (2022). Fibroblast Growth Factor 7 suppresses fibrosis and promotes epithelialization during wound healing in mouse fetuses. Int. J. Mol. Sci..

[43] Koike Y., Yozaki M., Utani A., Murota H. (2020). Fibroblast Growth Factor 2 accelerates the epithelial–mesenchymal transition in keratinocytes during wound healing process. Sci. Rep..

[44] Ortega S., Ittmann M., Tsang S.H., Ehrlich M., Basilico C. (1998). Neuronal defects and delayed wound healing in mice lacking fibroblast growth factor 2. Proc. Natl. Acad. Sci. USA.

[45] Jansen R.G., van Kuppevelt T.H., Daamen W.F., Kuijpers Jagtman A.M., Von den Hoff J.W. (2009). FGF2 loaded collagen scaffolds attract cells and blood vessels in rat oral mucosa. J. Oral Pathol. Med..

[46] Zakrzewska M., Marcinkowska E., Wiedlocha A. (2008). FGF 1: From biology through engineering to potential medical applications. Crit. Rev. Clin. Lab. Sci..

[47] Liu N., Guan S., Wang H., Li C., Cheng J., Yu H., Lin L., Pan Y. (2018). The antimicrobial peptide Nal P 113 exerts a reparative effect by promoting cell proliferation, migration, and cell cycle progression. BioMed Res. Int..

[48] Shin S.H., Koh Y.G., Lee W.G., Seok J., Park K.Y. (2023). The use of epidermal growth factor in dermatological practice. Int. Wound J..

[49] Wang X., Ji X. (2025). Application of recombinant human epidermal growth factor in oral and maxillofacial trauma and its impact on healing time. J. Stomatol. Oral Maxillofac. Surg..

[50] Tekin G.G., Deveci B., Deveci E. (2024). Ellagic acid protected the gingival tissue via fibroblast and epidermal growth factors in rats. Acta Cir. Bras..

[51] Sabbah D.A., Hajjo R., Sweidan K. (2020). Review on Epidermal Growth Factor Receptor (EGFR) Structure, Signaling Pathways, Interactions, and Recent Updates of EGFR Inhibitors. Curr. Top. Med. Chem..

[52] Rujirachotiwat A., Suttamanatwong S. (2021). Curcumin promotes Collagen Type I, Keratinocyte Growth Factor 1, and Epidermal Growth Factor Receptor expressions in the in vitro wound healing model of human gingival fibroblasts. Eur. J. Dent..

[53] Meizarini A., Aryati A., Rianti D., Riawan W., Puteri A. (2020). Effectivity of zinc oxide turmeric extract dressing in stimulating the re-epithelization phase of wound healing. Vet. World.

[54] Tang L., Cai S., Lu X., Wu D., Zhang Y., Li X., Qin X., Guo J., Zhang X., Liu C. (2024). Platelet-Derived Growth Factor Nanocapsules with Tunable Controlled Release for Chronic Wound Healing. Small.

[55] Illescas-Montes R., González-Acedo A., Melguizo-Rodríguez L., García-Recio E., Ruiz C., García-Martínez O., Ramos-Torrecillas J. (2025). Modulation of Gene Expression in Human Fibroblasts by Punicalagin and Ellagic Acid: An In Vitro Study. Mol. Nutr. Food Res..

[56] Piccin A., Di Pierro A.M., Canzian L., Primerano M., Corvetta D., Negri G., Mazzoleni G., Gastl G., Steurer M., Gentilini I. (2016). Platelet gel: A new therapeutic tool with great potential. Blood Transfus..

[57] Tavelli L., Ravidà A., Barootchi S., Chambrone L., Giannobile W.V. (2021). Recombinant human platelet-derived growth factor: A systematic review of clinical findings in oral regenerative procedures. JDR Clin. Trans. Res..

[58] Ahmad A., Nawaz M.I. (2022). Molecular mechanism of VEGF and its role in pathological angiogenesis. J. Cell Biochem..

[59] Bao P., Kodra A., Tomic Canic M., Golinko M.S., Ehrlich H.P., Brem H. (2009). The role of vascular endothelial growth factor in wound healing. J. Surg. Res..

[60] Shi Z., Yao C., Shui Y., Li S., Yan H. (2023). Research progress on the mechanism of angiogenesis in wound repair and regeneration. Front. Physiol..

[61] Keswani S.G., Balaji S., Le L.D., Leung A., Parvadia J.K., Frischer J., Yamano S., Taichman N., Crombleholme T.M. (2013). Role of salivary vascular endothelial growth factor (VEGF) in palatal mucosal wound healing. Wound Repair. Regen..

[62] Tanaka S., Yasuda T., Hamada Y., Kawaguchi N., Fujishita Y., Mori S., Yokoyama Y., Yamamoto H., Kogo M. (2020). Synthetic peptide SVVYGLR upregulates cell motility and facilitates oral mucosal wound healing. Peptides.

[63] Yamano S., Kuo W.P., Sukotjo C. (2013). Downregulated gene expression of TGFβs in diabetic oral wound healing. J. Craniomaxillofac. Surg..

[64] Bártolo I., Reis R.L., Marques A.P., Cerqueira M.T. (2022). Keratinocyte Growth Factor-Based Strategies for Wound Re-Epithelialization. Tissue Eng. Part. B Rev..

[65] Zheng D., Liwinski T., Elinav E. (2020). Interaction between microbiota and immunity in health and disease. Cell Res..

[66] Yuan H., Chlipala G.E., Bangash H.I., Meenakshi R., Chen D., Trivedi H.M., DiPietro L.A., Gajendrareddy P., Chen L. (2025). Dynamics of Human Palatal Wound Healing and the Associated Microbiome. J. Dent. Res..

[67] Engen S.A., Rørvik G.H., Schreurs O., Blix I.J., Schenck K. (2017). The oral commensal *Streptococcus mitis* activates the aryl hydrocarbon receptor in human oral epithelial cells. Int. J. Oral Sci..

[68] Hou K., Wu Z.-X., Chen X.-Y., Wang J.-Q., Zhang D., Xiao C., Zhu D., Koya J.B., Wei L., Li J. (2022). Microbiota in health and diseases. Sig Transduct. Target. Ther..

[69] Vanlancker E., Vanhoecke B., Sieprath T., Bourgeois J., Beterams A., De Moerloose B., De Vos W.H., Van de Wiele T. (2018). Oral microbiota reduce wound healing capacity of epithelial monolayers, irrespective of the presence of 5-fluorouracil. Exp. Biol. Med..

[70] Harasani Z., Ferdosi-Shahandashti E., Najafzadehvarzi H., Bakhshandeh B., Rajabzadeh A., Javadi K. (2025). In Vivo Evaluation of *Lacticaseibacillus reuteri* and *Lacticaseibacillus rhamnosus* Lysates for Oral Wound Healing. Probiotics Antimicrob. Proteins.

[71] Caselli E., Fabbri C., D’Accolti M., Soffritti I., Bassi C., Mazzacane S., Franchi M. (2020). Defining the Oral Microbiome by Whole-Genome Sequencing and Resistome Analysis: The Complexity of the Healthy Picture. BMC Microbiol..

[72] Han N., Jia L., Guo L., Su Y., Luo Z., Du J., Mei S., Liu Y. (2020). Balanced Oral Pathogenic Bacteria and Probiotics Promoted Wound Healing via Maintaining Mesenchymal Stem Cell Homeostasis. Stem Cell Res. Ther..

[73] Oudhoff M.J., van den Keijbus P.A., Kroeze K.L., Nazmi K., Gibbs S., Bolscher J.G., Veerman E.C. (2009). Histatins enhance wound closure with oral and non-oral cells. J. Dent. Res..

[74] Castro M., Torres P., Solano L., Córdova L.A., Torres V.A. (2019). Histatin-1 counteracts the cytotoxic and antimigratory effects of zoledronic acid in endothelial and osteoblast-like cells. J. Periodontol..

[75] Torres P., Díaz J., Arce M., Silva P., Mendoza P., Lois P., Molina-Berríos A., Owen G.I., Palma V., Torres V.A. (2017). The salivary peptide histatin-1 promotes endothelial cell adhesion, migration, and angiogenesis. FASEB J..

[76] Okuyama K., Yanamoto S. (2024). Saliva in balancing oral and systemic health, oral cancer, and beyond: A narrative review. Cancers.

[77] Ngeow W.C., Tan C.C., Goh Y.C., Deliberador T.M., Cheah C.W. (2022). A narrative review on means to promote oxygenation and angiogenesis in oral wound healing. Bioengineering.

[78] Li L., Ma Q., Mou J., Wang M., Ye J., Sun G. (2023). Basic fibroblast growth factor gel preparation induces angiogenesis during wound healing. Int. J. Artif. Organs..

[79] Pansani T.N., Basso F.G., Soares D.G., Hebling J., Costa C.A. (2016). Functional differences in gingival fibroblasts obtained from young and elderly individuals. Braz. Dent. J..

[80] Pansani T.N., Basso F.G., Turrioni A.P., Soares D.G., Hebling J., de Souza Costa C.A. (2017). Effects of lowlevel laser therapy and epidermal growth factor on the activities of gingival fibroblasts obtained from young or elderly individuals. Lasers Med. Sci..

[81] Tariq M., Tahir H.M., Butt S.A., Ali S., Ahmad A.B., Raza C., Summer M., Hassan A., Nadeem J. (2021). Silk-derived formulations for accelerated wound healing in diabetic mice. PeerJ.

[82] Sun J., Chen T., Zhao B., Fan W., Shen Y., Wei H., Zhang M., Zheng W., Peng J., Wang J. (2023). Acceleration of oral wound healing under diabetes mellitus conditions using bioadhesive hydrogel. ACS Appl. Mater. Interfaces.

[83] Radović K., Brković B., Roganović J., Ilić J., Milić Lemić A., Jovanović B. (2022). Salivary VEGF and postextraction wound healing in type 2 diabetic immediate denture wearers. Acta Odontol. Scand..

[84] Morishita Y., Hasegawa S., Koie S., Nakaya S., Goto M., Miyachi H., Naruse K., Nakamura N., Hayashi T., Kawai T. (2023). Effects of heated tobacco products and conventional cigarettes on dental implant wound healing: Experimental research. Ann. Med. Surg..

[85] Rathinavelu A., Aneed M.A. (2015). Tumor Angiogenesis and Novel Vascular Endothelial Receptor (VEGFR) Specific Small Molecule Inhibitors. Biopharmaceutical Drug Design and Development.

[86] Lassig A.A.D., Bechtold J.E., Lindgren B.R., Pisansky A., Itabiyi A., Yueh B., Joseph A.M. (2018). Tobacco exposure and wound healing in head and neck surgical wounds. Laryngoscope.

[87] Yang L., Song Y., Jiao Y., Liu S., Liu Y., Guo L., Liu Y. (2025). An Innovative Compound That Promotes Oral Wound Healing via Mobilizing Gingival Mesenchymal Stem Cell Homing. Biochem. Biophys. Rep..

[88] Budi H.S., Handajani J., Amir L.R., Soekanto S.A., Ulfa N.M., Wulansari S.A., Shen Y.K., Yamada S. (2025). Nanoemulgel Development of Stem Cells from Human Exfoliated Deciduous Teeth-Derived Conditioned Medium as a Novel Nanocarrier Growth Factors. Eur. J. Dent..

[89] de Farias G.A., Wagner V.P., Correa C., Webber L.P., Pilar E.F.S., Curra M., Carrard V.C., Martins M.A.T., Martins M.D. (2019). Photobiomodulation therapy modulates epigenetic events and NF-κB expression in oral epithelial wound healing. Lasers Med. Sci..

[90] Etemadi A., Taghavi Namin S., Hodjat M., Kosarieh E., Hakimiha N. (2020). Assessment of the Photobiomodulation Effect of a Blue Diode Laser on the Proliferation and Migration of Cultured Human Gingival Fibroblast Cells: A Preliminary In Vitro Study. J. Lasers Med. Sci..

